# Expected Medium- and Long-Term Impact of the COVID-19 Outbreak in Oncology

**DOI:** 10.1200/GO.20.00589

**Published:** 2021-02-02

**Authors:** Concetta Elisa Onesti, Marco Tagliamento, Giuseppe Curigliano, Nadia Harbeck, Rupert Bartsch, Hans Wildiers, Vivianne Tjan-Heijnen, Miguel Martin, Sylvie Rottey, Daniele Generali, Mario Campone, Massimo Cristofanilli, Lajos Pusztai, Marc Peeters, Guy Berchem, Javier Cortes, Thomas Ruhstaller, Eva Ciruelos, Hope S. Rugo, Guy Jerusalem

**Affiliations:** ^1^ Medical Oncology Department, CHU Sart Tilman Liege and Laboratory of Human Genetics, GIGA Research Institute, University of Liège, Liège, Belgium; ^2^ Oncology Department, University of Genova and IRCCS Ospedale Policlinico San Martino, Genova, Italy; ^3^ Oncology and Hemato-Oncology, European Institute of Oncology, IRCCS and University of Milan, Milan, Italy; ^4^ Breast Center, Department OB&GYN and CCCLMU, LMU University Hospital, Munich, Germany; ^5^ Department of Medicine I, Division of Oncology, Comprehensive Cancer Center, Medical University of Vienna, Wien, Austria; ^6^ Department of General Medical Oncology, University Hospitals Leuven, Leuven, Belgium; ^7^ Medical Oncology, Maastricht University Medical Center (MUMC), Maastricht, Netherlands; ^8^ Departamento de Medicina, Instituto de Investigación Sanitaria Gregorio Marañón Universidad Complutense, Madrid, Spain; ^9^ Department of Medical Oncology, UZ Gent, Gent, Belgium; ^10^ UO Patologia Mammaria e Ricerca Traslazionale—Breast Unit, Azienda Socio-Sanitaria, Territoriale di Cremona and University of Trieste, Cremona, Italy; ^11^ Medical Oncology, Institut de Cancérologie de l'Ouest-Pays de la Loire, Saint-Herblain, France; ^12^ Robert H. Lurie Comprehensive Cancer Center, Feinberg School of Medicine, Northwestern University, Chicago, IL; ^13^ Yale Cancer Center, Yale School of Medicine, New Haven, CT; ^14^ Oncology Department, University Hospital Antwerp (UZA), Edegem, Belgium; ^15^ Hemato-Oncology Department, Centre Hospitalier de Luxembourg, Luxembourg, Luxembourg; ^16^ Oncology Department, IOB Institute of Oncology, Quiron Group, Madrid, Barcelona, Spain; ^17^ Vall d'Hebron Institute of Oncology (VHIO), Centro Cellex, Carrer de Natzaret, Barcelona, Spain; ^18^ Medical Oncology, Breast Center Eastern Switzerland, St Gallen, Switzerland; ^19^ University of Basel, St Gallen, Switzerland; ^20^ Medical Oncology, University Hospital 12 de Octubre, Madrid, Spain; ^21^ Breast Care Center, University of California San Francisco Helen Diller Family Comprehensive Cancer Center, San Francisco, CA; ^22^ Medical Oncology, CHU Sart Tilman Liège and University of Liège, Liège, Belgium

## Abstract

**PURPOSE:**

The COVID-19 pandemic has affected healthcare systems globally, leading to reorganization of medical activities. We performed an international survey aimed to investigate the medium- and long-term impact on oncology units.

**MATERIALS AND METHODS:**

An 82-item survey was distributed from June 17 to July 14, 2020 among medical oncologists worldwide.

**RESULTS:**

One hundred nine medical oncologists from 18 countries in Europe (n = 93), United States (n = 5), and Latin America (n = 11) answered the survey. A systematic tracing of COVID-19–positive patients was continued in the postacute phase by 77.1% of the centers; 64.2% of the respondents participated in a local registry and 56% in international or national registries of infected patients. Treatment adaptations were introduced, and surgery was the most affected modality being delayed or canceled in more than 10% of patients in 34% of the centers, whereas early cessation of palliative treatment was reported in 32.1% of the centers; 64.2% of respondents reported paying attention to avoid undertreatments. The use of telemedicine has been largely increased. Similarly, virtual tools are increasingly used particularly for medical education and international or national or multidisciplinary meetings. 60.6% of the participants reduced clinical activity, and 28.4% compensated by increasing their research activity. Significant reduction of clinical trial activities is expected in 37% of centers this year. The well-being of healthcare staff would not recover by the end of the year according to 18% of the participants.

**CONCLUSION:**

The COVID-19 outbreak has had a major impact on oncologic activity, which will persist in the future, irrespective of geographical areas.

## INTRODUCTION

Since late 2019, a new coronavirus disease (COVID-19), caused by the severe acute respiratory syndrome coronavirus 2 (SARS-CoV-2), has been spreading worldwide and was declared as a pandemic on March 11, 2020.^1,2^

**CONTEXT**

**Key Objective**
The COVID-19 outbreak has had a significant and bursting influence on oncology units; how will this change oncologic care organization in the near future?
**Knowledge Generated**
The emergence of the pandemic has led to treatment adaptations, with surgery being the most affected modality, followed by chemotherapy and radiotherapy. Moreover, the health emergency has led to an increase in the use of telemedicine and virtual meetings and to the reduction of clinical trial activities.
**Relevance**
Many of the changes introduced in oncology to cope with the pandemic have a greater impact on the treatment of patients and the well-being of healthcare staff, and some of these will persist in the future, particularly all telemedicine activities.

The symptomatologic profile of COVID-19 is highly variable, from asymptomatic cases to severe syndromes, characterized by respiratory failure and organ injury.^3,4^ Recognized risk factors for severe symptomatology by SARS-CoV-2 infection are older age; male sex; poor performance status; and presence of comorbidities such as underlying pulmonary disease, cardiovascular disease, diabetes, and cancer.^5–7^ For patients with cancer, susceptibility to infection and the characteristics of COVID-19 vary depending on tumor type, stage of disease, and active treatments, with a higher risk of infection, complications, and case fatality for hematologic and lung malignancies.^6,8–11^ Considering this specific high-risk, emergency measures to control the spread of the infection were implemented at the peak of the pandemic.^12^ These included the use of treatment regimens with longer intervals, a preference for oral treatments instead of intravenous, the earlier discontinuation of palliative treatments, the use of hypofractionated radiotherapy instead of standard-course radiotherapy, and the expanded use of telemedicine.^13^ All these measures were principally based on expert opinions, in the absence of strong scientific evidence.^14^ In addition to oncologic care, other areas have been impacted by the COVID-19 pandemic, such as medical education, scientific activity, and the well-being of both patients and healthcare staff.^15–17^ These changes are likely to have short- and long-term impact.

With the purpose to analyze the impact on oncologic care organization, treatment decisions, scientific activity, medical education, and the well-being of healthcare workers, we performed this survey with broad representation from several countries.

## MATERIALS AND METHODS

An anonymous online questionnaire (Data Supplement) was sent by e-mail and shared through online platforms to oncologists (both specialists and in training) on June 17, 2020. The last answer was recorded on July 14. The majority of answers were mandatory, with the exception of questions regarding personal and psychologic well-being during the COVID-19 pandemic and questions on breast cancer care.

### Study Objectives

The current survey aimed at investigating the measures adopted to manage the COVID-19 outbreak in oncology departments, the measures implemented to reorganize clinical, research, and educational activities, and the short- and long-term impact of the pandemic on oncologic care, scientific activity, and well-being of healthcare workers.

### Characteristics of the Survey

This survey was designed by a working group of medical oncologists (n = 20) involved in clinical and research activities in different areas. It was composed of 82 questions, which were divided into different sections: (1) demographic, medical training, and employment information of respondents (questions 1-9); (2) experience with COVID-19 in the oncology departments and preventive measures adopted (questions 10-20); (3) data collection of COVID-19 and outcome of patients with cancer who developed COVID-19 disease (questions 21-22); (4) COVID-19 impact on oncologic treatments in general, with a focus on breast cancer (questions 23-37); (5) teleconsultations (questions 38-48); (6) activity in oncology departments (questions 49-51); (7) virtual meetings (questions 52-65); (8) clinical activity and scientific production during COVID-19 crisis (questions 66-71); (9) COVID-19 and psychologic impact (questions 72-78); and (10) COVID-19 and clinical trials (questions 79-82).

### Statistical Analysis

Considering the descriptive nature of the study, no sample size calculation was preplanned. We estimated a target population of survey recipients to be approximately 400 oncologists. With an expected margin of error of 8% with a 95% confidence level, 100 respondents would be needed.

Analyses were mainly descriptive. To explore differences in categorical variables in answers collected regarding the same issue during the acute phase and the postacute phase of the COVID-19 infection course, a McNemar test was applied. A Fisher's exact test was applied to evaluate categorical variables. Descriptive analyses were conducted to investigate potential differences in the answers provided by oncologists working in Europe compared with those working in the United States and Latin America. Tests were two-sided, and *P* < .05 was considered statistically significant.

## RESULTS

The 82-item survey was distributed by 20 medical oncologists from ten of the most affected countries during the period June 17 to July 14, 2020. A total of 109 physicians from 18 countries filled out the survey. The median age of participants was 48.5 years (interquartile range, 38 to 55.8), with a median of 20 years (interquartile range, 10.5 to 25) working in oncology including residency; the majority were male (61.5%). Sixty-eight participants (62.4%) worked in academic hospitals, 32 (29.6%) in community hospitals, and five (4.6%) in private centers without overnight ward. The place of work was in most cases a general hospital with oncology unit (n = 72, 66.1%) or a cancer center (n = 35, 32.1%). The main oncology subspecialization was breast cancer for the majority (60.6%). Table 1 summarizes respondents' characteristics.

**TABLE 1:**
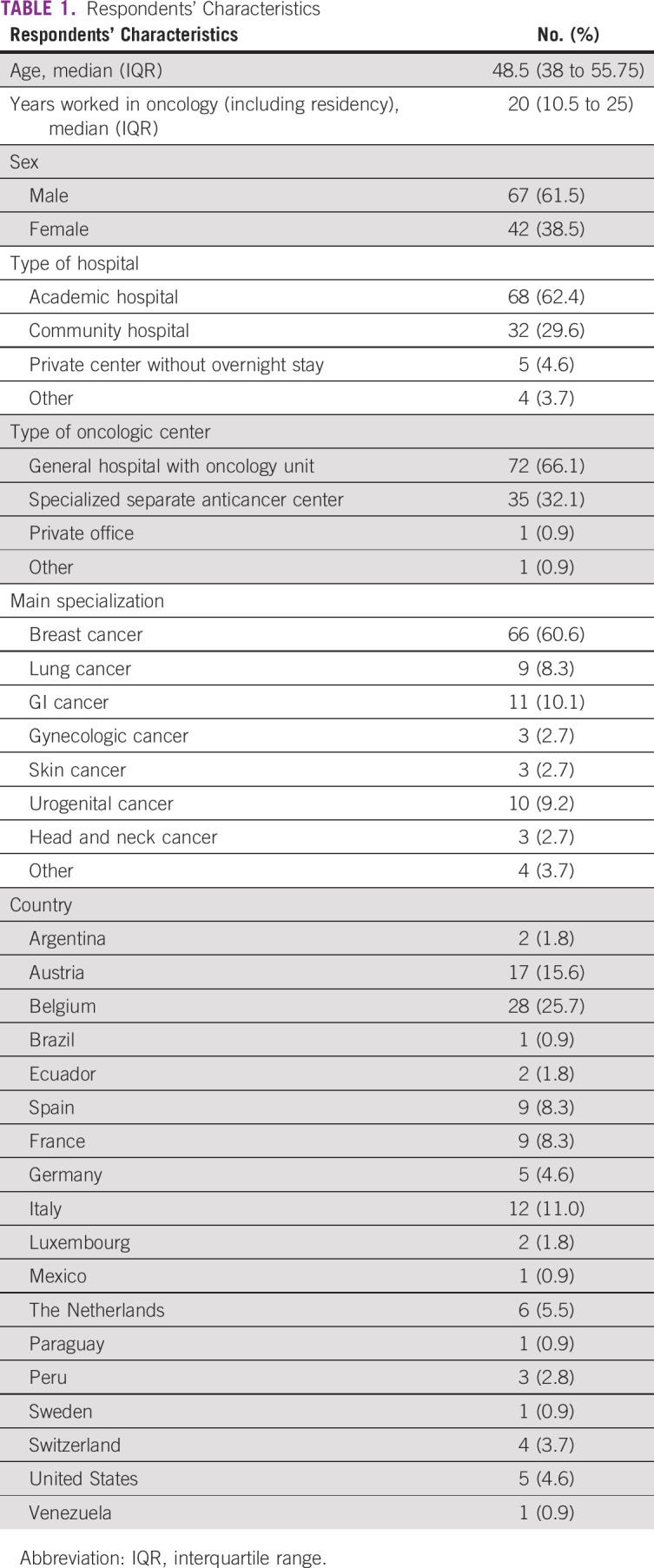
Respondents' Characteristics

During the survey, the participating countries had different infection loads, with an incidence risk ranging from 8.7 new cases/100,000 inhabitants in Italy to 383.4/100,000 in the United States (Table 2).^18,19^ In seven countries (Austria, Belgium, France, Germany, Italy, the Netherlands, and Spain) the survey was completed after the first peak (n = 86), in four countries (Luxembourg, Sweden, Switzerland, and United States) during a resurgence of the pandemic (n = 12), and in seven countries (Argentina, Brazil, Ecuador, Mexico, Paraguay, Peru, and Venezuela) at the beginning of the outbreak (n = 11).

**TABLE 2:**
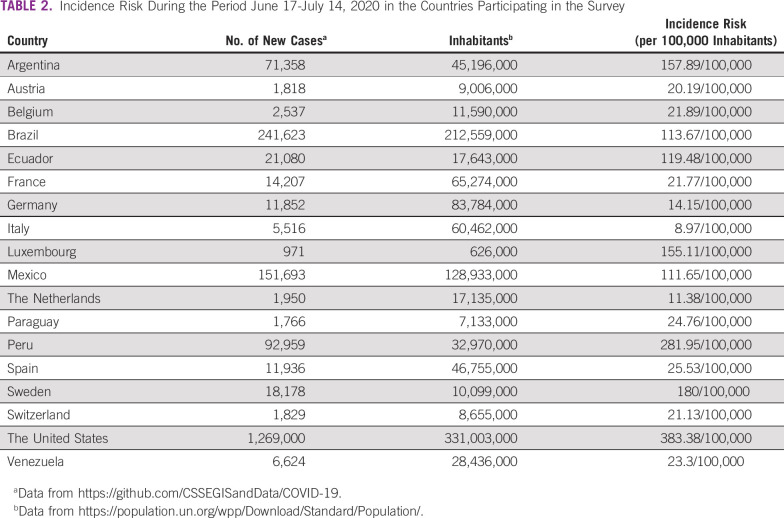
Incidence Risk During the Period June 17-July 14, 2020 in the Countries Participating in the Survey

### Experience With COVID-19 and Data Collection

**FIG 1:**
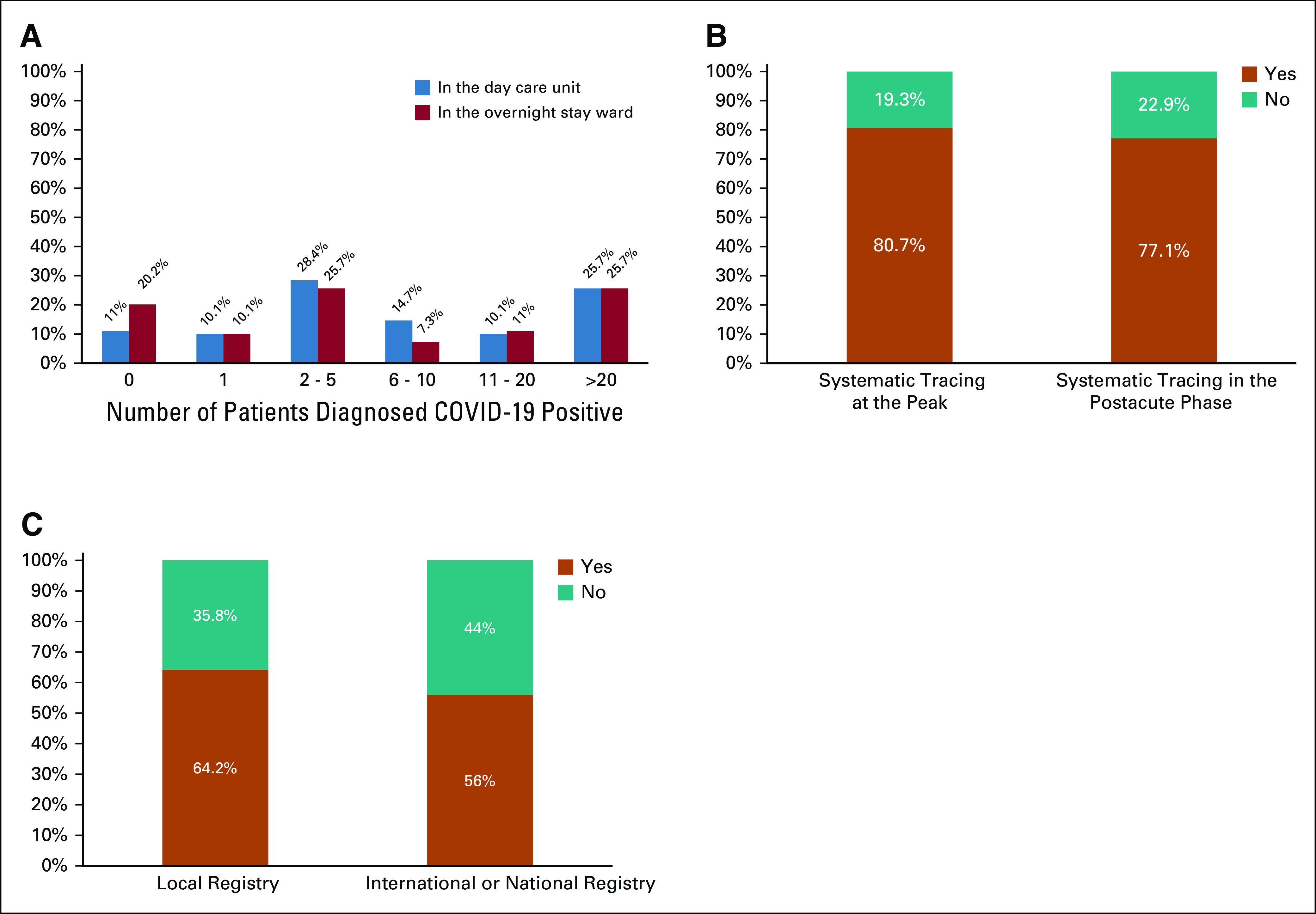
Experience with COVID-19 and data collection. (A) The number of cases diagnosed with COVID-19 in the participants' centers in the day care unit and in the overnight stay ward. Answers concerning systematic tracing and participation in local or national or international registry (B and C, respectively).

Figure 1A summarizes the cumulative number of patients diagnosed with COVID-19 at each site at the time of survey completion. At the peak of the pandemic, 80.7% of the centers organized a systematic tracing of infected patients, and 77.1% extended the systematic tracing in the postacute phase (*P* = .125) (Fig 1B). A local registry of infected patients was organized in 64.2% of the cases, and 56% of the respondents participated in an international or national registry (Fig 1C). No significant differences were observed between Europe, United States, and Latin America.

### Monitoring of COVID-19 Infection

A triage for COVID-19 infection was done with nasal or throat swab or gargle test before admission to day care unit and to overnight stay and before starting a new treatment in 32.1%, 62.4%, and 27.5% of the participants' centers during the peak of the pandemic and in 27.5%, 58.7%, and 30.3% in the postacute phase, respectively. The differences between the use of nasal or throat swab or gargle test as the triage method during and after the peak of the pandemic were not significant (*P* value for day care unit, overnight stay ward, and new treatment of .06, .12, and .25, respectively); no significant differences were observed by geographic area as well.

Serologic tests were performed before initiating a new systemic anticancer therapy in only 7.3% of the cases as routine and in 12.8% as part of research. Interestingly, the use of serologic test before starting a new treatment was significantly higher in Latin America (36.4%) compared with Europe (4.3%) and the United States (0%), although small sample size limits this analysis.

### COVID-19 Impact on Oncologic Treatment

The majority (64.2%) of respondents agreed that the most important risk for oncology patients is undertreatment by stopping or adapting the therapy, rather than the risk of dying from COVID-19.

**FIG 2:**
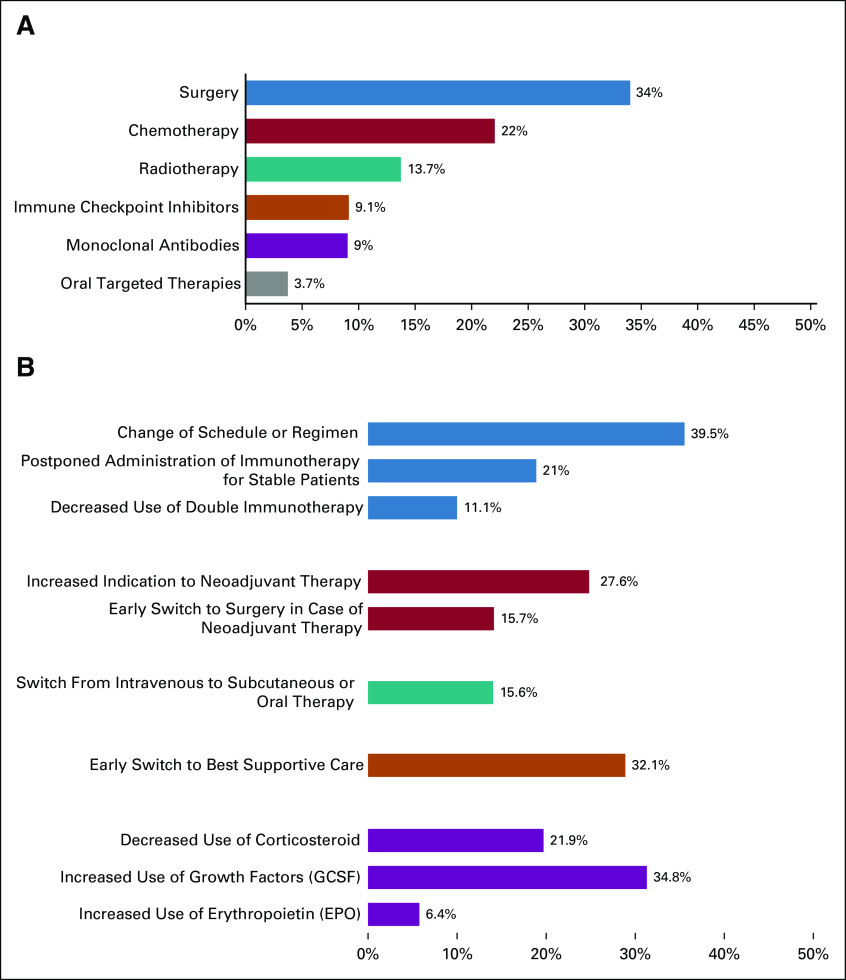
Treatment modalities affected by COVID-19 pandemic. (A) The treatment modalities affected by COVID-19 pandemic are summarized. (B) The types of modifications of treatment modalities are shown in detail. In both the figures, the percentages represent the rate of changes observed in more than 10% of the patients by the respondents to the survey. EPO, erytropoietin; G-CSF, granulocyte colony-stimulating factor.

Surgery was the most affected therapy being canceled or delayed in more than 10% of patients in 34% of the centers, followed by chemotherapy in 22%, radiotherapy in 13.7%, immunotherapy in 9.1%, antibody treatment in 9%, and oral targeted therapy in 3.7% (Fig 2A). However, 15.7% of the respondents reported earlier progression to surgery in patients receiving neoadjuvant therapy (> 10%, Fig 2B), whereas 27.6% noted increased use of neoadjuvant systemic therapy instead of primary surgery in more than 10% of patients. Regarding radiotherapy, standard fraction treatment was more frequently affected than stereotactic radiotherapy, with a rate of cancellation or delay in more than 10% of patients by 13.7% and 8.3% of the centers, respectively. Conversely, 42.2% of the participants did not report delay or discontinuation of oral targeted therapy. Therapies not affected were immunotherapy in 40.4% of the participants' centers, monoclonal antibody treatment in 39.4%, stereotactic radiotherapy in 38.5%, standard fraction radiotherapy in 27.7%, chemotherapy in 19.3%, and surgery in 12.8%.

In breast cancer (89 respondents), the most affected treatment was everolimus, which was estimated to be permanently stopped or delayed in more than 10% of patients by 15%, followed by CDK4/6 inhibitors by 8.9% and alpelisib by 6.7% of respondents.

A switch from intravenous to subcutaneous or oral formulation was reported in more than 10% of patients by 15.6% of the participants (Fig 2B). To reduce hospital admissions, an increased use of home administration was reported by 51.6% of the participants. Overall, 18.3% of the participants stated that their center already performed home deliveries before the COVID-19 outbreak, which increased to 45% during the peak of the pandemic and to 34.9% in the postacute phase.

**FIG 3:**
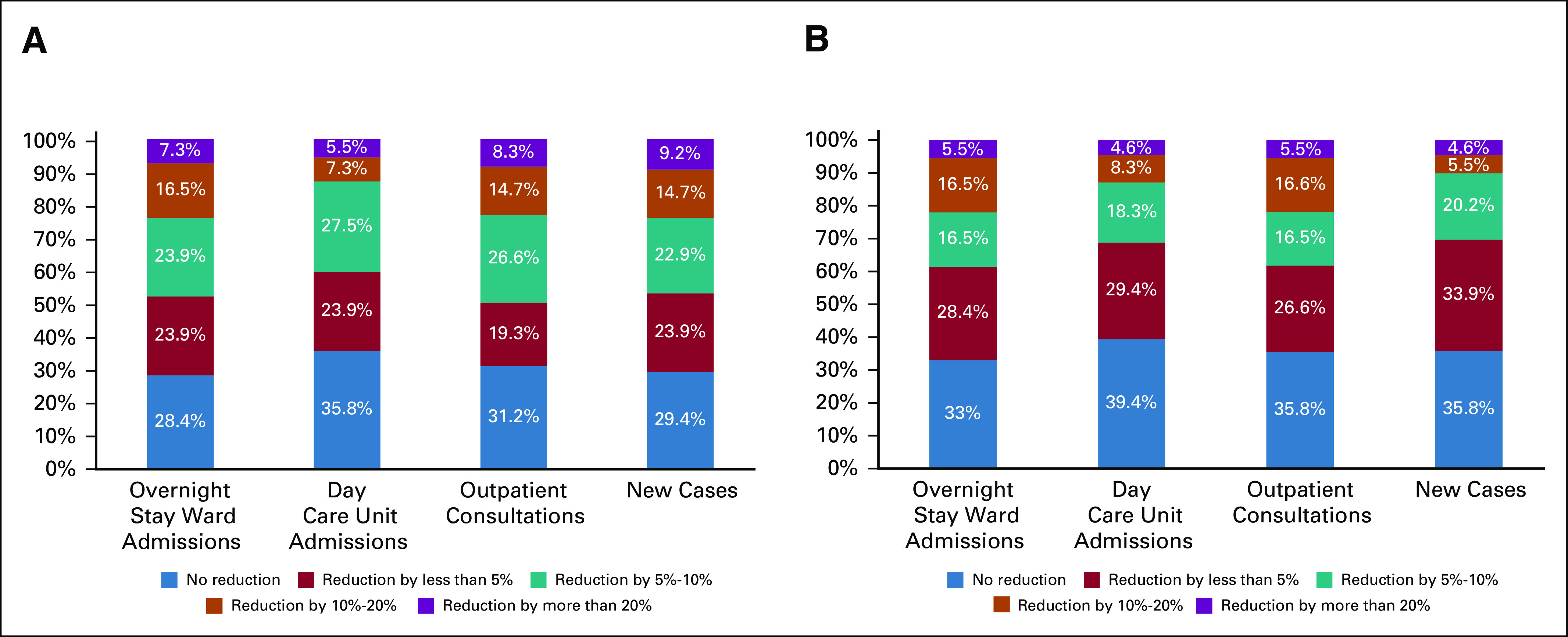
Reduction of activity in oncology departments because of COVID-19 outbreak. Magnitude of the reduction of activity in oncology departments in June-July 2020 (A) and the expected reduction at the end of 2020. The percentages showed represent the proportion of participants in the survey.

Treatment adaptations were introduced, including a change of schedule or regimen in more than 10% of patients by 39.5% of the respondents to the survey, postponement of immunotherapy for stable patients by 21%, and the decreased use of double immunotherapy by 11.1% (Fig 3B). In addition, modification of concomitant treatments was considered, such as decreased use of corticosteroids and increased use of granulocyte colony-stimulating factor (G-CSF) and erythropoietin in more than 10% of patients, respectively, by 21.9%, 34.8%, and 6.4% of the survey respondents (Fig 2B).

An early switch to best supportive care was also reported. In particular, 77.1% of the participants noted early cessation of systemic treatment in at least one patient and 32.1% in more than 10% of patients (Fig 2B).

Decreased oncology unit hospitalizations and use of emergency units was observed in at least one patient by 81.6% and 81.7% of the participants, respectively, and in more than 10% of patients by 44.9% and 52.3%.

At the time of the survey, most participants (73.4%) stated that treatment choice was no longer influenced by COVID-19. A significant difference was found by geographical area, with 78.5% of the European respondents stating that treatment choice was no longer influenced by COVID-19 versus 40% in the United States and 54.5% in Latin America (*P* = .01), reflecting the difference in the severity of the pandemic at the moment of survey completion.

### Activity of Oncology Departments

The activity of oncology clinics during the COVID-19 crisis significantly decreased compared with that prior to the pandemic. Particularly, 69.7% of the participants stated that the number of consultations decreased, whereas working days increased in length according to 45.9% of the participants, because of the prolongation of each visit.

The activity load in June-July 2020 was reduced in terms of admissions to the overnight stay ward, admissions to the day care unit, outpatient consultations, and the number of new cases compared with the overall experience before the COVID-19 crisis in 71.6%, 64.2%, 68.9%, and 71.6% of the respondents' centers (Fig 3A). The margin for improvement in the clinical activity load expected by the end of 2020 was minimal. In fact, a decrease in admissions to the overnight stay ward, admissions to the day care unit, outpatient consultations, and the number of new cases were expected by 66.9%, 60.6%, 65.2%, and 64.2% of the respondents to the survey (Fig 3B).

### Teleconsultations and Virtual Meetings

**FIG 4:**
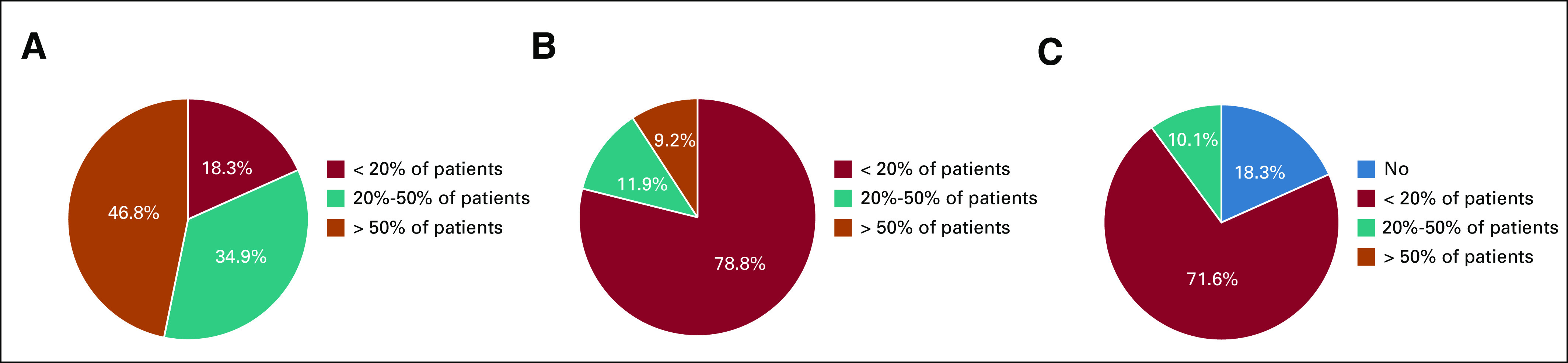
Teleconsultations during and after COVID-19 outbreak. The use of teleconsultations in oncology department during the peak of the pandemic (A) and in the postacute phase (B). (C) Representation of the expected use of telemedicine in the near future after COVID-19 crisis.

During the pandemic peak, a considerable increase in the use of telemedicine was observed. Teleconsultations by video or phone instead of on-site consultations were done for more than 20% of patients by 81.7% of the participants, compared with only 21.1% of the participants in the postacute phase (*P* < .0001) (Figs 4A and 4B). 16.5% of the physicians responding to the survey will not organize a new outpatient consultation for each visit managed by phone or video call during the COVID-19 crisis. The majority of the participants (81.7%) stated that they will continue to use telemedicine more in the future than in the past (Fig 4C).

During the pandemic peak, teleconsultations were done for patients in long-term follow-up (94.5%), for patients receiving oral drugs (92.7%), for patients receiving immunotherapy (57.8%), and for patients receiving chemotherapy (55%).

Nevertheless, regulatory issues still persist, without reimbursement for teleconsultations in 30.3% of the participating centers. Many regulatory barriers have been temporarily removed to accelerate the use of telehealth services during the pandemic; reimbursement for teleconsultations increased from 33% before COVID-19 to 69.7% during the crisis (*P* < .0001), although with a 25% reduction in fees compared with on-site consultation in about a quarter of the cases. Interestingly, an increase in reimbursement for telemedicine was reported only in Europe (from 29% to 69.9%, *P* < .0001) and in the United States (from 60% to 100%, *P* = .250), whereas in Latin America, teleconsultations were even reimbursed before the COVID-19 pandemic in 54.5% and the frequency of reimbursement remains unchanged.

To reduce interpersonal contacts, most meetings are taking place virtually. Virtual meetings were a good alternative to live meetings for multidisciplinary team for 61.5% of the medical oncologists, for administrative and/or strategic meetings for 63.3%, for continued medical education for 66.9%, for personal scientific discussions for 61.4%, for teaching for 43.2%, for international meetings for 33.9%, and for networking with industry and academic colleagues for 44.1%. Continued use of virtual meetings in all fields was expected by more than 80% of the participants.

### Scientific Activity and Clinical Trials

Although 60.6% of the participants reduced their clinical activity at the peak of the pandemic, 48.6% were unable to use the time saved for scientific activities because of the consequences of COVID-19 and the increased burden of organizing patient care. Only 28.4% of the participants reported an increase in scientific activity.

Laboratory activities were also interrupted during the peak of the pandemic according to 55.9% of the participants, and only 34% stated that laboratory activities had now resumed to the same level as before COVID-19.

Overall, 60.6% of the participants observed a reduction in clinical trial activity during the peak of the pandemic, as recommended by many regulatory authorities. For those clinical trials that continued, an increase in major protocol deviations was observed by 27.5% of the participants. Currently, clinical trial activities are open or will be reactivated in the near future according to 72.5% of the participants, in particular according to 76.3% of respondents from Europe, 40% from the United States, and 45.5% from Latin America (*P* = .07). A significant reduction in clinical trial activities is expected by the year end by 36.7% of the participants and 35.8% expect severe financial problems for their clinical trial unit.

### Psychologic Impact of COVID-19 Outbreak

The well-being of medical oncologists was explored by this survey with optional questions, answered by 108 (99.1%) participants. Overall, 50% stated that their general well-being was largely impacted at the peak of the pandemic; it is still impacted in the postacute phase in 29% and 18% expect not to recover to baseline by the end of the year.

Psychologic support for caregivers was organized by the institution of 62.7% of the participants, but only 10.3% have asked or have intention to ask for this service.

## DISCUSSION

The impact of COVID-19 on almost all medical activities has been widely documented and discussed. This includes outcome in patients with solid and hematologic cancers who become infected by SARS-CoV-2, modifications in oncologists' attitudes and decision making, and reorganization of oncologic care and facilities to reduce the risk for inhospital contagion and virus spread.^12,13,15,20,21^ What is more difficult to assess are the indirect consequences of the COVID-19 pandemic with regard to oncology, such as the reduced number of new cancer diagnoses during the acute phase of the epidemic because of the delay or interruption of cancer screening procedures.^22,23^ The projections about an expected cumulative excess of avoidable cancer deaths within the next years can only be estimated by modeling studies.^24,25^ To allow for a comprehensive overview on this subject, our survey aimed at investigating, to our knowledge, for the first time, the expected medium- and long-term burden of the COVID-19 pandemic on oncology, through the direct report of representatives from oncology departments throughout Europe, the United States, and Latin America. Our survey confirms that COVID-19 has had a major impact on organization of patient care, well-being of caregivers, continued medical education, and clinical trial activities in oncology.

A systematic monitoring of the COVID-19 dynamics using nasal or throat swab or gargle test will be continued in the majority of centers, especially as a triage procedure before overnight hospitalization or surgery, reflecting an expected continuation of the pandemic in the near future. Of course, anticancer treatments have been readapted as suggested by various scientific societies and international guidelines, especially those that put the patient in a condition of greater immunodepression and/or have overlapping side effects with COVID-19 symptoms, albeit 64.2% of respondents agree or strongly agree that undertreatment is the most important risk for patients with cancer. This is in agreement with a recent study that showed similar COVID-19–related mortality rates and complications in patients with or without cancer, cautioning about possible consequences of treatment limitation in patients with cancer.^26^ From our survey emerged that surgery was the treatment modality most affected by COVID-19–related changes. Sud et al^27^ estimated that more than 4,700 deaths/year in England could be attributed to a 3-month delay to surgery across different cancers with stage 1-3 (with differences according to the tumor type). A systematic review revealed moderate evidence that a delayed surgical approach to GI malignancies can lead to a worse outcome, whereas another study did not show such a relationship for prostate cancers.^28,29^ The risks of tumor progression by delaying radical surgery should be evaluated on a case-by-case basis. In some diseases, such as luminal early breast cancer, simple clinical algorithms can help to delay surgeries without compromising patient outcome.^30^

Regarding systemic oncologic adaptation, it is worthy of consideration that 34.6% of respondents increased the use of G-CSF during the COVID-19 pandemic. Both the administration and inhibition of G-CSF are currently being under evaluation as therapeutic options for treating COVID-19 manifestations, thus its wide prophylactic use cannot be suggested lightly.^31^

Approximately 82% of participants estimate that they will continue to use telemedicine, and it is encouraging to find that coverage of its cost goes in parallel with the increase in its use.

Although the oncologic healthcare workforce hopes for a rapid normalization of professional activities after the lockdown, a long-term impact of COVID-19 is expected. Thus, the risk of delayed diagnosis of new cancers and economic consequences of COVID-19 on access to health care and cancer treatments have to be carefully evaluated. It is of concern that the need to obtain new scientific evidence to guide clinical practice in the next months may be hindered by reduced clinical trial and laboratory activities, although research activities seem to be recovering.

Moreover, the rapid spread of COVID-19 epidemics is associated with psychologic impact on oncology patients, compounded by multiple factors: knowledge that the individual is at higher risk of serious complication if infected by COVID-19, loneliness and isolation as a result of social distancing, and the underlying constant fear of the cancer.

The results of our international survey reflect the current status in international oncology but need to be interpreted with caution because of some intrinsic limitations. As stated above, the results are influenced by two main variables:

1. The epidemiology of COVID-19 infections in each country, in terms of the number of cases or inhabitants and timeline of the epidemic.
2. the strategies adopted by that country to face the pandemic.

To overcome these limitations, we have provided sub-analyses according to geographical areas. We grouped European countries together since almost all had < 30 COVID-19 cases/100,000 inhabitants at the time of the interview and were experiencing the first postpeak phase of the epidemic. For differences in the resources to cope with the epidemic, we did not consider United States and Latin America together, although some countries had similar contagion dynamics.

There are still many challenges ahead in the fight against COVID-19, with many unanswered questions facing practicing oncologists on a daily basis. Reflecting on real-world experience with data collected in the large registries that have been created will also help us extrapolate and determine which is the best evidence to be used in managing future cases of COVID-19 outbreaks or other pandemics. Differences in the prevalence of COVID-19 cases, clinical settings, staffing, and access to resources will result in varying adoption of many of these recommendations. Collective wisdom and experience from different organizations, communities, and governments will help shape the best approach to control the pandemic at a global level.

To conclude, the adaptations to a changed daily working life reflect the plasticity of oncology, in its nature accustomed to renewing itself to follow the continuous changes led by clinical research. It is important to deepen the understanding of indirect effects of the COVID-19 pandemic on organizational issues and the cost-effectiveness of diagnostic and treatment options, which can be more difficult to weigh but more crushing in the long run.
